# Current-induced giant polarization rotation using a ZnO single crystal doped with nitrogen ions

**DOI:** 10.1038/srep12762

**Published:** 2015-08-06

**Authors:** Naoya Tate, Tadashi Kawazoe, Wataru Nomura, Motoichi Ohtsu

**Affiliations:** 1 Kyushu University, 744 Motooka, Nishi-ku, Fukuoka 819-0395, Japan; 2 Nanophotonics Engineering Organization, 1-20-10, Sekiguchi, Bunkyo-ku, Tokyo 112-0014, Japan; 3 The University of Tokyo, 2-11-16 Yayoi, Bunkyo-ku, Tokyo 113-8656, Japan

## Abstract

Giant polarization rotation in a ZnO single crystal was experimentally demonstrated based on a novel phenomenon occurring at the nanometric scale. The ZnO crystal was doped with N^+^ and N^2+^ ions serving as p-type dopants. By applying an in-plane current using a unique arrangement of electrodes on the device, current-induced polarization rotation of the incident light was observed. From the results of experimental demonstrations and discussions, it was verified that this novel behavior originates from a specific distribution of dopants and the corresponding light–matter interactions in a nanometric space, which are allowed by the existence of such a dopant distribution.

Oxide semiconductors are widely used direct-transition materials having a large bandgap. Due to their natural abundance, innocuousness, and transparency to visible light, they are expected to be widely employed for fabricating various electrical and optical devices^1^,^2^,^3^,^4^,^5^,^6^,^7^,^8^,^9^,^10^. Although such oxide semiconductors are highly promising from the viewpoint of social needs, it is technically difficult to implement electro- or magneto-induced optical functions using the standard doping methods used with other types of semiconductors, because acceptors from the dopants are generally compensated with donors from the numerous oxygen vacancies and interstitial metals in the crystal. According to recent research by the authors’ group^11^,^12^,^13^,^14^,^15^,^16^,^17^,^18^,^19^, novel electro-optical characteristics using various indirect- and direct-transition semiconductors have been successfully demonstrated by employing our original annealing method which exploits the characteristic behavior of dressed photons (DPs)^20^. A technique known as DP-assisted annealing has been used to autonomously form a dopant distribution that works as an appropriate p–n homojunction layer to effectively induce DPs by light irradiation. Specifically, by using an n-type bulk zinc oxide (ZnO) crystal, which is one of the most common oxide semiconductors, a p–n homojunction-structured LED that emits light at room temperature has been successfully realized^12^. One of the fundamental points of this method is that the dopants form not only a spatially homogeneous distribution necessary for higher electrical conductivity but also nanometrically-clustered structures, which are autonomously optimized for generating DPs in their surroundings. According to recent theoretical discussions and experimental demonstrations by the authors’ group^21^,^22^,^23^,^24^,^25^, DPs were generated by irradiating light that is strongly coupled with phonons in the material, so that characteristic light–matter interactions occur via these DPs.

Magneto-optical effects are one of the most commonly known aspects of light–matter interactions. In particular, in the effect known as Faraday rotation, while light propagates through a specific material, that is, a magneto-optical material, under a large magnetic field, the polarization of the light rotates. Polarization rotation is a fundamental technology for several applications utilizing the polarization degree-of-freedom, such as ellipsometry and optical computing. The former is based on precise measurement and analysis of the polarization of reflected light from a target material, which requires a sophisticated polarization rotator. In particular, in recent research on ellipsometry, there has been a strong need for two-dimensional polarization rotators that can perform dynamic polarization modulation^26^,^27^,^28^. In optical computing, particularly the previously proposed polarization-encoded optical computing method^29^,^30^, advanced polarization rotators are required for processing. Furthermore, more recently, advanced polarization rotators are essential elements for realizing optical quantum computing and related research^31^,^32^,^33^, which is based on polarization-encoding of photons.

In the work described in this paper, we focused on a ZnO device as a polarization rotator. In this device, the dopant distribution is expected to be suitable for inducing polarization modulation due to the characteristic behavior of DPs. The ZnO device was fabricated by using the DP-assisted annealing method, and by means of a unique electrode arrangement, different from the arrangement normally used for LEDs, the device demonstrated novel functionality as a polarization rotator. The device was irradiated with linearly polarized light in the vertical direction, and extremely large polarization rotation of the incident light was observed. The phenomenon is quite similar to conventional magneto-optical effects, but exhibited a much higher level. In addition it does not require any external equipment for applying a strong electric or magnetic field, nor a long light propagation length for ensuring a sufficient interaction time for the modulation. We quantitatively evaluated the performance of our ZnO device when functioning as a novel optical rotator, and we discuss the origin of this phenomenon on the basis of DPs.

## Basics

Generally, an n-type crystal is doped by implanting p-type dopants during the crystal growth process. Previously, several techniques for doping ZnO crystals with p-type dopants using nitrogen have been demonstrated. These techniques have been applied to various methods of growing ZnO crystals, such as molecular beam epitaxy (MBE)^34^,^35^, chemical-vapor deposition (CVD)^36^,^37^,^38^, and magneto-sputtering^39^,^40^,^41^,^42^. However, in the case where oxide semiconductors such as ZnO are used, the self-compensation effect^43^ between acceptors and donors and dissipation of dopants has been still fundamental issues to achieve favorable p-type doping method.

In this study, an n-type bulk ZnO crystal was implanted with N^+^ and N^2+^ ions serving as p-type dopants *after* the growth process using the hydrothermal growth method^12^. According to previous reports by the authors’ group^11^,^12^,^13^,^14^,^15^,^16^,^17^,^18^,^19^, novel electro-optical functionalities in various types of indirect-transition semiconductors have been successfully implemented. The unique characteristics of these functionalities are fundamentally due to a specific distribution of dopants used for generating DPs in each material. A DP is a quasi-particle which represents the coupled state of a photon and an electron^25^. A DP excites a coherent phonon in a nanometric material, and the state of the DP is coupled with the states of the excited phonon. During the electron transition process in the material, this coupled state behaves as an intermediate state; hence, multistep transitions and corresponding optical functions are allowed. In order to realize effective generation of DPs, in our previous work, the device was subjected to DP-assisted annealing to optimize the dopant distribution for generating DPs; namely, they were annealed with Joule heating due to the application of a current while the device was irradiated with light having a lower photon energy than the bandgap energy of the material. The basics of the DP-assisted annealing method are explained below.

First, while Joule heat is generated by the application of a forward bias current to the device, the dopants try to randomly diffuse in the material to converge to a homogeneous distribution. However, at the same time, DPs tend to be generated by irradiation of light in the vicinity of the exciton polaritons. Here, the generation of DPs is due to the formation of localized electrons and holes at regions of a specific distribution of the dopants. In the case where the photon energy of the irradiated light is lower than the bandgap energy of the material, as in our method, the photon energy is not absorbed in the material and can reach the distribution of dopants, where it generates DPs. The generated DPs, which behave as localized energy fields^21^,^22^,^23^,^24^,^25^, can excite multimode coherent phonons in the material, and a coupled state of the DPs and phonons can be excited; in other words, a multi-step electron transition occurs via energy levels corresponding to the coupled state. Consequently, part of the energy due to Joule heat is converted to the photon energy of stimulated emission via the DPs, which is associated with the multi-step transition. Subsequently, Joule heat is lost and diffusion of the dopants necessarily stops. Now, the distribution of dopants works as an appropriate structure for effective generation of DPs. On the other hand, other dopants where DPs are hardly generated in their vicinities continue to diffuse in the material until they form the required specific distribution, and subsequent stimulated emission occurs. Finally, a high DP generation efficiency is exhibited in all regions, and the temperature drops in all regions of the device. More detailed theoretical descriptions of the DP-assisted annealing method and related achievements have been reported in previous papers by authors’ group^11^,^12^,^13^,^14^,^15^,^16^,^17^,^18^,^19^.

## Experiment

**Figure 1:**
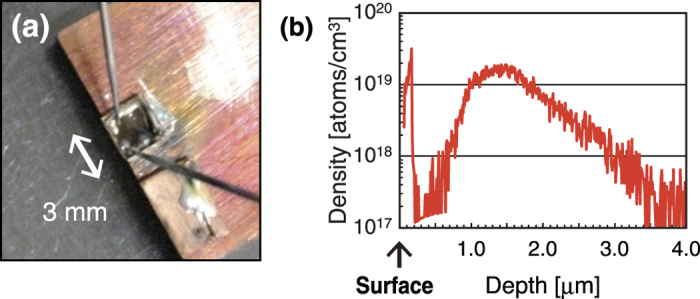
(**a**) Photograph of the fabricated ZnO device. (**b**) The dopant density versus implantation depth in the device.

In order to use the ZnO device as a polarization rotator, input light whose polarization direction was perpendicular to the crystal axis of the material was made incident on the device. Here, an original arrangement of electrodes was prepared for applying an in-plane current to the device. Details of the fabrication of the device and discussion of mechanism of the polarization rotation are given in the Methods and Discussion sections, respectively. Figure 1(a) shows a photograph of the fabricated ZnO device. The dopant implantation depth was confirmed by secondary ion mass spectrometry (SIMS) to be about 2 μm, as shown in Fig. 1(b). In a related study, the detailed dopant distribution formed by the DP-assisted annealing has been previously confirmed in the case of Si by using a three-dimensional atom probe (3DAP) method^17^.

**Figure 2:**
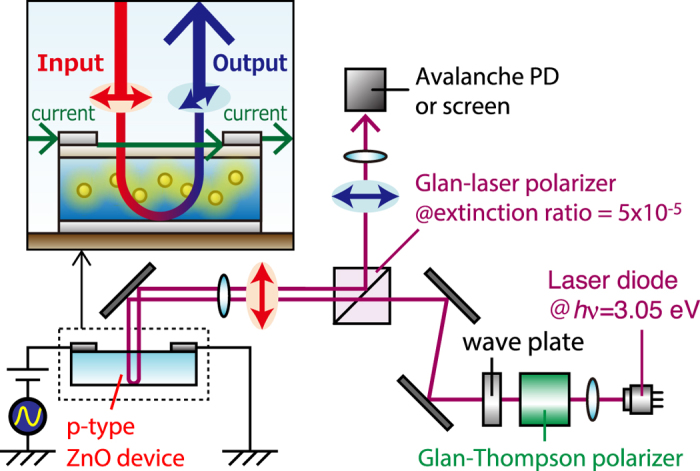
Schematic diagram of setup used for observing polarization rotation based on cross-Nicol method.

The experimental setup used for demonstrating polarization rotation is shown in Fig. 2. The setup was constructed to observe modulated light among the incident linearly-polarized light. The important point is that, as shown in the inset of Fig. 2, a current flowed in the in-plane direction of the device via surface electrodes formed of an indium tin oxide (ITO) layer. The applied voltage was sinusoidally modulated by a function generator. Linearly-polarized light having a photon energy *hν* = 3.05 eV, to which the device is transparent, was radiated in the vertical direction onto the device and was output in the opposite direction by reflection at the back side of the device. During propagation in the device, the incident light was expected to be affected by the applied current via interactions between the material and DPs. The mechanism is briefly discussed in the next section. As a result, corresponding polarization rotation was observed as a variation in the optical intensity of the reflected light by using a Glan-laser polarizer.

**Figure 3:**
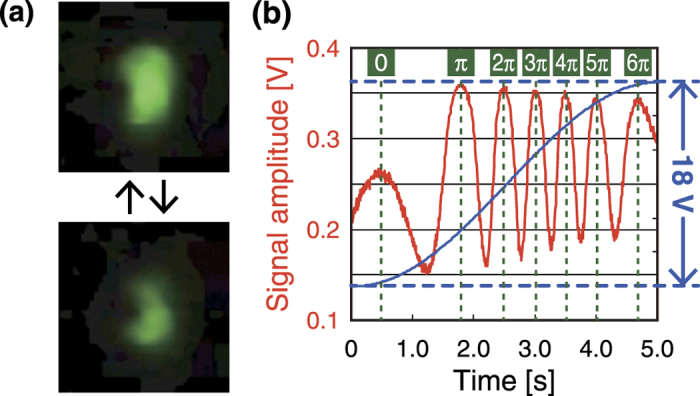
(**a**) Modulated output images, which were projected on a phosphor screen. (**b**) Experimentally observed relations between output light intensity (red line) and voltage applied to the device (blue line), where the maximum voltages were 18 V.

Figure 3(a) shows the modulated output projected on a phosphor screen, which was set at the output port. In order to clearly represent the spatial dependency of the modulation, the input light was radiated onto the device using an off-focus setup. Therefore, one cycle of varying brightness, between bright and dark parts at each area, corresponds to modulation of π radians. As shown, the spatial modulation of the polarization was found to depend on the applied current.

To quantitatively evaluate the polarization rotation shown in Fig. 3(a), input light was focused onto the device, and the intensity of the output light was observed by using an avalanche photodiode (PD). Fig. 3(b) shows an example of the output light intensity observed with 100 mHz sinusoidal modulation of an 18 V applied voltage. As shown, spatial modulation of the output signal amplitude was clearly observed. Importantly, Fig. 3(b) shows that, while the applied voltage was increased to 18 V during a half cycle of applied voltage, the polarization of the input light was rotated by more than 6π radians.

## Discussion

**Figure 4:**
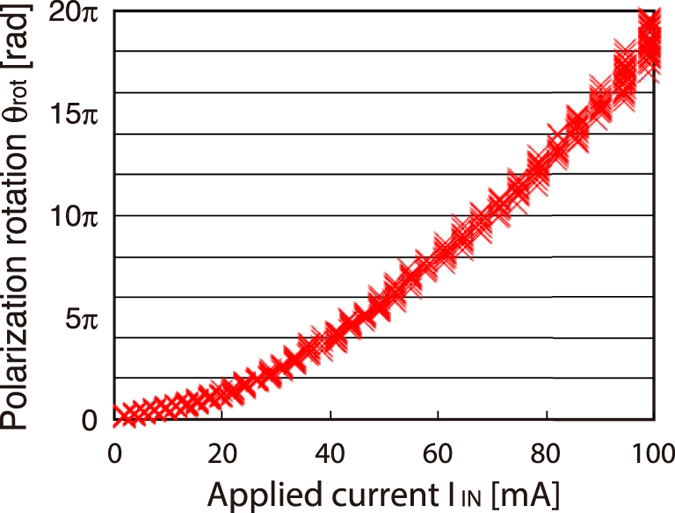
Relation between applied current *I*_IN_ and polarization rotation *θ*_rot_.

In order to explain the large amount of polarization rotation achieved by our device, the relation between the applied current *I*_IN_ and the polarization rotation *θ*_rot_ is plotted by using the measured I-V relation of the device. As shown in Fig. 4, the device achieved a polarization rotation of more than 20π radians by applying 100 mA of current. By description with amounts of Faraday rotation, it reveals at intermediate between dielectric semiconductors and magnetic metals.

The important point of our approach is that such a giant polarization rotation does not require any external equipment for applying a strong magnetic field, unlike the conventional magneto-optical effect. Also, it does not require a long propagation distance of the incident light; the effective propagation distance for the modulation was less than 5 μm, because of the back and forth propagation in the device, as indicated by the SIMS results shown in Fig. 1(b). The basic mechanism of such a large polarization rotation is discussed below.

Simple and direct propagation of light in the material under a current-induced magnetic field is hardly expected to reveal such an effect, and therefore, the involvement of DPs must be considered. Namely, incident light induces DPs in the surroundings of the dopants, and, at the same time, current-induced magnetic fields strongly affect the phase of the DPs via the distribution of dopants. In such a situation, the incident optical energy and magnetic fields are allowed efficiently interact with each other via the DP states, where the DP states are efficiently energetically coupled with the phonon states of the material. After the interaction, reconversion of optical energy from the DPs to propagating light occurs, and other interactions are induced in surroundings of other dopants. While the expected amount of modulation in a single process is not large enough, after a number of iterations of this process, finally a large amount of modulation is realized. We expect that the theoretical relation between the phase of the DPs and magnetic fields will be clarified by future experiments.

**Figure 5:**
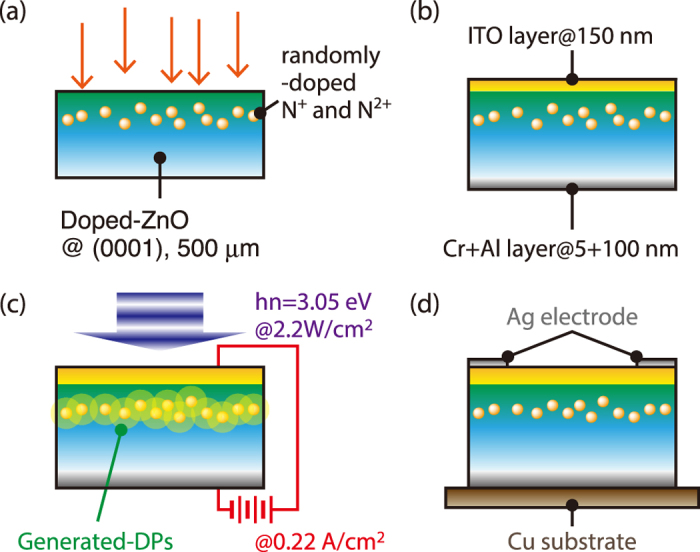
Basic process for fabricating a ZnO device. (**a**) Implantation of N^+^ and N^2+^ ions into n-type ZnO single crystal. (**b**) Depositing electrode layers at the front side and back side of the crystal. (**c**) Application of forward bias current and irradiation of light for the DP-assisted annealing. (**d**) Deposition of Ag electrode on the crystal and bonding of crystal to Cu substrate for heat dissipation.

Additionally, practical specifications for use as an optical device, namely, switching speed and power consumption, are theoretically estimated as follows: From the size of the surface (5 mm × 5 mm) and the thickness (500 μm), our ZnO device is estimated to have a capacitance of 30 pF, which will require 6 nC ( =30 pF/500 μm) of electric charge to achieve a single operation, namely π radians of polarization rotation. By referring to the results of our experimental demonstration shown in Fig. 5, if 10 mA of current is assumed to be applied, a switching speed of 120 ns ( =6 nC/50 mA) is expected. On the other hand, energy of 60 nJ ( =6 nC × 10 V) is required for a single operation.

## Conclusion

In the work described in this paper, giant polarization rotation was experimentally demonstrated using a ZnO single crystal doped with N^+^ and N^2+^ ions. This giant polarization rotation is fundamentally due to an optimized distribution of dopants in the ZnO crystal achieved by DP-assisted annealing, which allowed DPs to be effectively induced by incident light, and application of an in-plane current to the device by using a unique electrode arrangement. As a result, polarization rotation of more than π/3 radian per volt, which is the amount required in practice to implement optical rotation, was successfully observed without using any external equipment for applying a large magnetic field, and with an effective light propagation distance of less than 5 μm in the device. Based on the results of demonstrations, we considered that this giant polarization rotation was fundamentally due to the repeated interactions between current-induced magnetic fields and localized DPs generated in the regions around dopants in a nanometric space.

## Methods

Preparation of ZnO device: To fabricate the prototype device, we used commercially available n-type ZnO single crystal with a thickness of 500 μm, which is prepared by the hydrothermal growth method^44^. The crystal axis orientation was (0001), and the initial electrical resistivity of the crystal was 50–150 Ω cm. The basic process for fabricating a junction ZnO device is schematically shown in Fig. 5.

N^+^ and N^2+^ ions were implanted into the crystal in six steps at energies of 20, 50, 100, 200, 400, and 600 keV for producing a broad distribution of dopants. The ion dose densities were set to 4.20 × 10^13^, 9.00 × 10^13^, 2.25 × 10^14^, 2.75 × 10^14^, 4.50 × 10^14^, and 4.50 × 10^14^ cm^−2^, respectively (Fig. 5(a)). By using multi-step implantation with these parameters, the implanted dopants were distributed in a broad region in the crystal to form a p-type layer. Then, using radio-frequency sputtering, a 150 nm-thick ITO layer was deposited on the front side of the sample, and a 5 nm-thick Cr layer and a 100 nm-thick Al layer were deposited on the back side of the sample to serve as electrode layers (Fig. 5(b)). After deposition, DP-assisted annealing was performed (Fig. 5(c)). During Joule heating by application of a forward bias current, the device was irradiated with laser light having a photon energy *hν* = 3.05 eV, which is lower than the bandgap energy of the ZnO crystal (3.40 eV). The forward bias current density was set to 0.22 A/cm^2^, which was as high as possible without causing thermal destruction of the device, and the power of the irradiated light was set to 2.2 W/cm^2^, which was as high as possible without causing the crystal temperature to become too high. According to the previous work by the authors’ group^12^, the surface temperature rose to nearly 100 °C under similar experimental conditions, and then dropped to a constant temperature 70 °C. As we described in the Basics section, this drop in temperature corresponds to the generation of DPs, bringing about stimulated emission, and indicates successful completion of the annealing process for fabricating an appropriate distribution of dopants to efficiently generate DPs in the material. Then, Ag electrodes were deposited for applying an in-plane current to the device, in a direction perpendicular to direction during the annealing process. Finally, the crystal was bonded on a Cu substrate for heat dissipation while working as a polarization rotator (Fig. 5(d)). The Cr and Al layers were utilized as a reflecting surface for the incident light applied to the device.

## Additional Information

**How to cite this article**: Tate, N. *et al.* Current-induced giant polarization rotation using a ZnO single crystal doped with nitrogen ions. *Sci. Rep.* **5**, 12762; doi: 10.1038/srep12762 (2015).
